# Comparisons of microbiological characteristics and antibiotic resistance of *Klebsiella pneumoniae* isolates from urban rodents, shrews, and healthy people

**DOI:** 10.1186/s12866-020-1702-5

**Published:** 2020-01-14

**Authors:** Xue-shan Zhong, Yong-zhi Li, Jing Ge, Gang Xiao, Yun Mo, Yu-qi Wen, Jing-ping Liu, Yi-quan Xiong, Min Qiu, Shu-ting Huo, Ming-ji Cheng, Qing Chen

**Affiliations:** 1 Department of Epidemiology, School of Public Health, Southern Medical University, Guangzhou, 510515 China; 2 Department of clinical Laboratory, the Third Affiliated Hospital of Southern Medical University, Guangzhou, 510630 China

**Keywords:** Urban rodent, Shrew, *Klebsiella pneumoniae*, Antimicrobial resistance, Hypervirulence, PFGE, MLST

## Abstract

**Background:**

The comparisons of molecular characterization and antibiotic resistance of *Klebsiella pneumoniae* (KP) isolates from humans and other animal hosts are not well studied. Our goal was to compare the molecular epidemiology of KP strains that were isolated from urban rodents, shrews, and healthy people.

**Results:**

*K. pneumoniae* (KP) isolates were isolated from fecal samples of rodents, shrews and healthy adults in 2015 in southern China. In total, 465 fecal samples were collected, of which 85 from rodents, 105 from shrews, and 275 from healthy adults. Antimicrobial susceptibility and production of extended-spectrum β-lactamases (ESBL) of the isolates were tested. PCR-based methods were used to detect specific genes, including ESBL genes (*bla*_TEM_, *bla*_SHV,_ and *bla*_CTX-M_) in ESBL-producing isolates, capsular serotypes (K1, K2, K5, K20, K54, and K57) in hypervirulent KPs (hvKPs), and virulence genes (*magA*, *wcaG*, *rmpA*, *uge*, *kfu*, and *aerobactin*) in hvKP isolates. Multilocus sequence type (MLST) and pulsed-field gel electrophoresis (PFGE) were performed to exclude the homology of these isolates. The carriage rate of KP in urban rodents and shrews (78.42%) was higher than that in healthy adults (66.18%) (*χ*^2^ = 8.206, *P* = 0.004). The prevalence rates of ESBL-producing isolates among rodents, shrews, and humans were 7.94, 12.79, and 17.03%, respectively. The positive rates of CTX-M, TEM and SHV types in ESBL-producing isolates were 29.79, 27.66, and 17.02%, respectively. Serotype K1, K5, K20, and K57 were detected in both small mammals and humans. PFGE typing revealed thirty-six clusters. PFGE cluster A was clustered by samples of shrews and healthy adult, with a similarity of 88.4%. MLST typing revealed thirty-eight types. ST23 and ST35 were detected in samples of shrews and healthy adults. ST37 was detected in samples of 2 rodents and a healthy adult.

**Conclusions:**

Overlapping serotypes of hvKP were observed in both the animals and humans. The same PFGE or MLST types were also found in isolates derived humans, rodents and shrews. Therefore, urban rodents and shrews might play a certain role in the transmission of drug-resistant and hypervirulent KP.

## Background

*Klebsiella pneumoniae* (KP) is ubiquitous in humans, animals, sewage, soil, and polluted waters [1]. KP is considered an important cause of community-acquired (CA) pneumonia infections. Since the early 1970s, KP has been detected in hospital environments and has become a leading cause of hospital-acquired (HA) infections, such as nosocomial pneumonia, wound, soft tissue, or urinary tract infections in neonates, the elderly, and immuno-deficient patients [2–4]. Recently, life-threatening cases of CA infections, including pyogenic liver abscesses, meningitis, and endophthalmitis, caused by KP have been reported in the general population [5].

Efficiency of colonization and the ability to acquire resistance to antibiotics enables KP to spread rapidly in healthcare centers [4]. With the emergence of multidrug-resistant (MDR) strains associated with hospital outbreaks, MDR KP is becoming an urgent threat to public health [6]. The main molecular mechanism of drug resistance in KP is the production of extended-spectrum β-lactamases (ESBL) [7]. In the early 1980s, ESBL were first identified in KP and *Serratia marcescens* strains in Europe [8]. Since then, ESBL-producing KP has become widespread to a lager extent [9]. ESBL are plasmid-mediated enzymes that hydrolyze oxyimino-β lactam agents, such as third-generation cephalosporins and aztreonam [10]. These plasmids can also carry resistance genes to other antibiotics including fluoroquinolones, cotrimoxazole, and aminoglycosides [7].

In addition, a new type of virulent variant KP, known as hypervirulent KP (hvKP), which is associated with community-acquired infections, has emerged worldwide over the past three decades. HvKP infects both the healthy and non-immuno-compromised people more easily, and has more significant morbidity and mortality than the “classic” KP [5]. Infections caused by KP have also been described in cases from various animals in veterinary studies, including companion animals, horses, cattle, birds, monkeys, elephants, seals, guinea pigs, and rats [11, 12].

Urban rodents and shrews have long share living environments with humans. These small mammals may play a role as reservoirs of causative agents for various bacterial, viral, and parasitic zoonoses because of their pervasiveness and their propensity toward close contact with humans [13]. Rats have been previously reported to carry multidrug-resistant bacteria such as *Escherichia coli* [14, 15], *Staphylococcus aureus* [16], and *Salmonella* spp. [17]. Additionally, some species of shrews in urban areas have been found to carry several bacterial pathogens of zoonotic diseases, such as *Bartonella* spp. [18] and *Leptospira* spp. [19]. Thus, urban rodents and shrews are important reservoirs of rodent-borne diseases in cities. Increasing urbanization and poverty have resulted in the emergence or re-emergence of rodent-associated diseases in urban areas [13]. Therefore, it is necessary to understand the prevalence and microbiological characteristics of zoonotic pathogens circulating among these small mammals in urban environments. To the best of our knowledge, there has yet to be studies on the prevalence and microbiological characteristics of KP carried by urban rodents and shrews.

The objectives of this study were to understand the carriage rate of KP in urban rodents and shrews, and to characterize antimicrobial resistance and hyper virulence of KP in these small mammals from a community in southern China. Meanwhile, KP isolates from healthy adults in the community were compared with those from small mammals. Based on these results, we assessed the potential transmission among urban rodents, shrews, and humans in a community.

## Results

### Sample collection and bacterial isolation

Between May and September of 2015, a total of 190 rodents and shrews, including 80 *Rattus norvegicus*, 3 *Mus musculus*, 2 *Rattus flavipectus*, and 105 *Suncus murinus* (Asian house shrews), were captured. The seasonal distribution of the capture rates was mainly in June and July (Additional file 1: Figure S1). Among the 190 small mammals, KP was isolated from 149 (78.42%) individuals, including 63 *Rattus norvegicus* and 86 Asian house shrews. None of *Mus musculus* and *Rattus flavipectus* was positive for KP. There was no significant difference in KP carriage rates between *Rattus norvegicus* (74.12%) and house shrews (81.90%) (*χ*^2^ = 0.288, *P* = 0.591) (Additional file 2: Table S4).

A total of 275 stool samples from healthy adults were collected during the same period. The detection rate for KP was 66.18% (182/275). The carriage rate of KP in the small mammals was higher than that in healthy adults (78.42% vs. 66.18%, *χ*^2^ = 8.206, *P* = 0.004) (Additional file 2: Table S4).

### Antimicrobial susceptibility

As shown in Table 1, the antimicrobial susceptibility patterns of 331 KP isolates from the small mammals and healthy adults were similar. All isolates had a low level of susceptibility to penicillin and cephalosporin antibiotics. Susceptibility rates to piperacillin for rodents, shrews, and healthy adults were 6.35, 13.95, and 19.23%, respectively. All isolates had low susceptibility to cefazolin, which were less than 5%. For the third-generation cephalosporin, susceptibility rates to cefotaxime were 34.92, 37.21, and 35.16%, respectively; susceptibility rates to ceftazidime were 66.67, 63.95, and 65.38%, respectively. Susceptibility rates to cefepime, which is one of the fourth-generation antibiotics, were 79.37, 59.30, and 54.40% for rodents, shrews, and healthy adults, respectively.

**Table 1:** Antibiotic susceptibility patterns of KP isolates from urban rodents, shrews, and healthy people in 2015

Antibiotics	Rodents (*n* = 63)			Shrews (*n* = 86)			Healthy adults (*n* = 182)		
	S (%)	I (%)	R (%)	S (%)	I (%)	R (%)	S (%)	I (%)	R (%)
piperacillin	6.35	52.38	41.27	13.95	48.84	37.21	19.23	40.66	40.11
cefazolin	3.17	46.03	50.79	3.49	45.35	51.16	2.20	46.70	51.10
cefuroxime	6.35	90.48	3.17	4.65	93.02	2.33	4.95	92.31	2.75
cefotetan	0	3.21	96.79	1.20	4.70	94.20	1.58	2.22	96.20
cefotaxime	34.92	50.79	14.29	37.21	39.53	23.26	35.16	36.81	28.02
ceftazidime	66.67	25.40	7.94	63.95	29.07	6.98	65.38	23.08	11.54
cefepime	79.37	19.05	1.59	59.30	38.37	2.33	54.40	44.51	1.10
aztreonam	95.24	1.59	3.17	93.02	1.16	5.81	92.31	5.49	2.20
meropenem	1.63	26.93	71.44	0	23.30	76.70	0	13.20	86.80
norfloxacin	100.00	0.00	0.00	98.84	0.00	1.16	96.70	1.10	2.20
ciprofloxacin	92.06	6.35	1.59	95.35	3.49	1.16	91.76	5.49	2.75
amikacin	98.41	1.59	0.00	100.00	0.00	0.00	98.90	0.55	0.55
gentamicin	96.83	0.00	3.17	95.35	1.16	3.49	96.15	0.00	3.85
chloramphenicol	84.13	4.76	11.11	94.19	0.00	5.81	89.01	0.55	10.44
tetracycline	79.37	3.17	17.46	84.88	4.65	10.47	75.82	1.65	22.53
trimethoprim-sulfamethoxazole	84.13	1.59	14.29	84.88	5.81	9.30	72.53	13.19	14.29
amoxicillin-clavulanate	87.30	9.52	3.17	88.37	8.14	3.49	80.22	18.68	1.10

*S* Susceptible, *I* Intermediate, *R* Resistant

### ESBL-producing and multidrug-resistant KP

The positive rates of ESBL-producing isolates were 7.94, 12.79, and 17.03% in rodents, shrews, and healthy adults, respectively. The multi-drug resistance rates for rodents, shrews, and healthy adults were 49.21, 36.04, and 47.80%, respectively. There was no statistically significant difference among rodents, shrews, and healthy adults (Additional file 2: Table S5).

Among all ESBL-producing isolates, the prevalence of CTX-M, TEM and SHV types were 29.78% (14/47), 27.65% (13/47) and 17.02% (8/47), respectively (Additional file 2: Table S5).

### Capsular serotyping and virulence genes

As shown in Additional file 2: Table S6, 20 hvKP isolates of 6 common serotypes were identified among 331 KP isolates. All six serotypes were detected in isolates from healthy adults. Serotype K1, K5, K20, and K57 were detected in both the small mammals and the healthy adults. Among all hypervirulent isolates, the *magA* gene was only present in K1 isolates, whereas the *uge* gene was detectable in all serotypes. All K1 isolates and some K20, K54, and K57 isolates carried the *wcaG* gene. The *Aerobactin* gene was identified among all *rmpA*-positive isolates. All K2 isolates lacked the *kfu* gene while the other five serotypes were positive for the *kfu* gene. As shown in Additional file 2: Table S6, K1, K2, K20, and K57 isolates carried more of these six virulence genes than did the K5 and K54 isolates.

### ESBL-producing KP fingerprinting

**Fig. 1:**
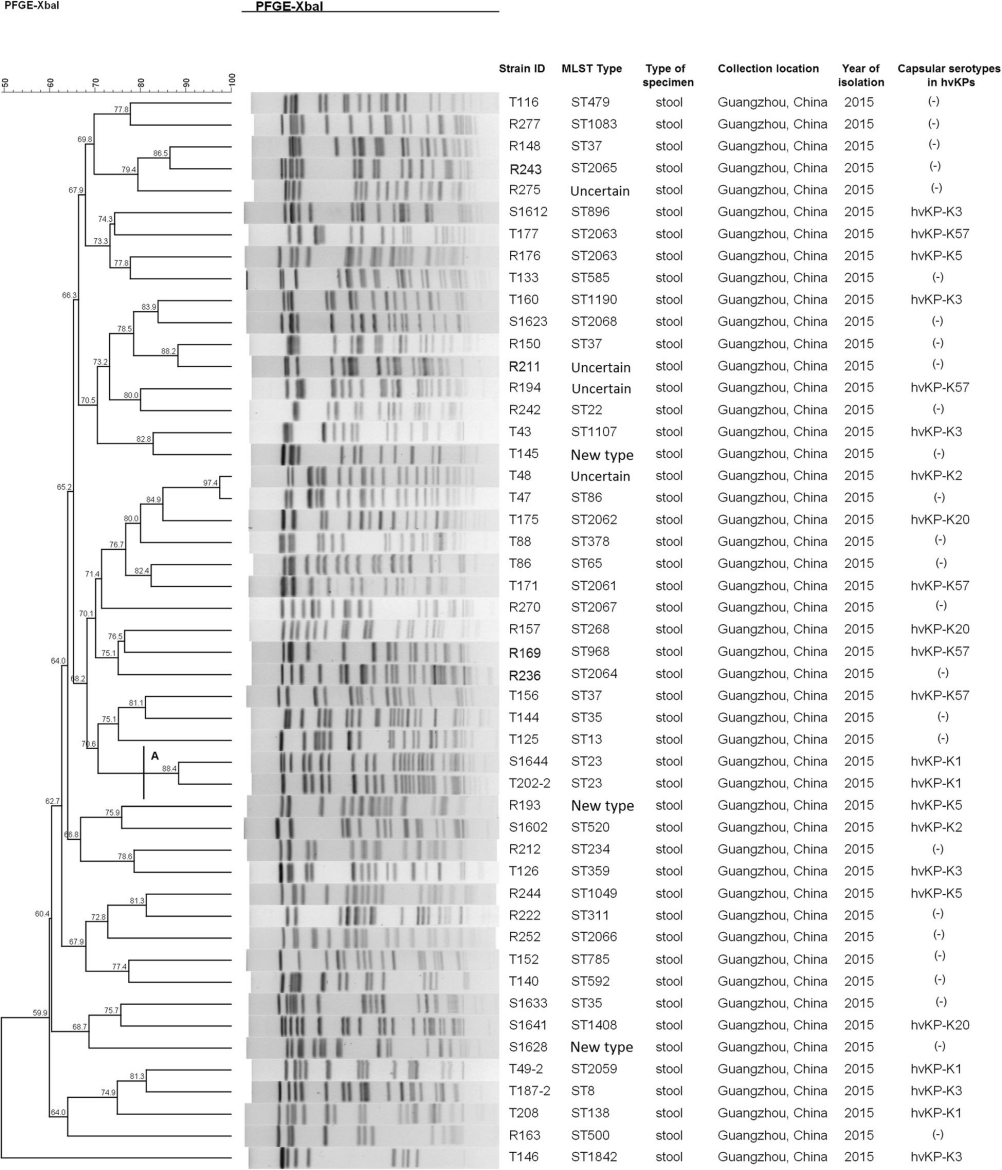
Relationships of the ESBL-producing KP isolates based on Pulsed Field Gel Electrophoresis (PFGE). A PFGE pattern with more than 80% DNA bands that are different from the others is taken to be a unique PFGE pattern (S for samples of shrews; R for samples of rodents; T for samples of healthy adults; For MLST type, uncertain means lacking of one housekeeping gene data, new type means no matched MLST type from the website)

Forty-seven ESBL-producing KP isolates which were no direct epidemiological association showed 36 PFGE types. PFGE type A clustered by samples of a shrew(S1644) and a healthy adult(T202–2). Whose similarity of the PFGE pattern was up to 88.4% (Fig. 1). The ESBL-producing KP isolates demonstrated 38 MLST types. ST23 was detected in samples of a shrew(S1644) and a healthy adult(T202–2). Likewise, ST35 were detected in samples of a shrew(S1633) and a healthy adult(T144). ST37 belonged to samples of rodents (R148, R150) and a healthy adult(T156) (Fig. 1).

Among them, because of the isolates being not collected in an outbreak investigation of KP, PFGE profile only showed partial similarity of the sample sets. Interestingly, the similarity of isolates of T48 and T47, R148 and R243, R150 and R211 were more than 85%, respectively (Fig. 1). But each two samples of which were the same type of host that collected during the different time period, suggesting that the potential transmission may be exist among urban rodents, shrews, and humans in a community.

## Discussion

### Carriage prevalence

*K. pneumoniae* can be asymptomatically present in the gastrointestinal tract, eyes, respiratory tract, and genitourinary tract of healthy humans [3]. In most infections with KP, colonization in the gastrointestinal tract seems to be a prerequisite for the establishment of infections [20]. Carriage rates of KP varied considerably among different populations. In European populations, the carrier rate of KP in fecal samples from healthy people ranged from 10.1 to 35.7% [21, 22]. In Asian countries, the carriage rates of KP from fecal specimens varied, which was from 18.8 to 75.0% [23, 24]. In our study, the fecal carriage rate of KP from healthy adults (66.18%) was similar to that of healthy Chinese residents in Asian countries, except Japan. Rats and shrews are known as a source of zoonotic pathogens responsible for significant human morbidity and mortality. Their feces are considered an important pathway for disseminating common bacteria through direct or indirect interactions with humans, food, water or sewage systems [13]. In our study, the fecal carriage rate of KP among these small mammals was significantly higher than that of healthy adults.

### Antibiotic resistance

The emergence of KP antibiotic susceptibility, especially to third and fourth generation cephalosporins, is a critical concern for the development of treatments against bacterial infection [25]. A recent global surveillance database collected from Europe, North and South America, and Asia, showed that the detection rates for ESBL-producing KP were 7.5–44% [26]. In China, 30.1–39.3% of the total isolates collected from hospital or community acquired infections during 2003–2013 period were ESBL-positive [27]. In our study, we identified 7.94 and 12.79% of KP isolated from rodents and shrews to be ESBL-producers, the ESBL-positive rate among healthy adults was higher than that in studies conducted in Hungary [28, 29]. Notably, there was no significant difference in the ESBL-positive rate among urban rodents, shrews, and healthy adults.

### Capsular serotypes

Six serotypes of KP, including K1, K2, K5, K20, K54, and K57, are frequently considered important hypervirulent serotypes, which have been not only associated with nosocomial infections in immunocompromised patients, but also the cause of life-threatening community-acquired infections, such as liver abscess in healthy individuals, especially in Asian countries [30–32]. The results of our study showed that all six serotypes of hvKP were identified among healthy adults, and K1, K2, and K57 serotypes were predominant. This finding is consistent with the previous investigation that K1 and K2 serotypes were the predominant types of hvKP among healthy Chinese in Asian countries [24]. Serotype K57 has emerged as frequent cause of septic arthritis, blood infection, urinary tract infections, and respiratory tract infection, but was rarely reported among healthy adults [30, 33]. However, we had observed the high prevalence of K57 serotype isolates among healthy adults. Data on the serotype distribution of hvKP in fecal samples from urban rodents and shrews have rarely been reported previously. Among all hypervirulent isolates from these small mammals, the current study showed high prevalence of serotype K5 and K57, which were prevalent among community onset infections, including community acquired pneumonia, nasopharynx, urinary tract infection, blood infection, pyogenic liver abscess and so on [30, 34–36].

Several cases of multidrug-resistant hvKP-related hospital-acquired infections have been reported [37–39]. In our study, we found resistance to penicillin and cephalosporin antibiotics among hvKP isolates, especially in the K5, K20, and K57 serotype isolates from healthy adults and small mammals. The antimicrobial resistance patterns of KP isolates from urban rodents, shrews, and healthy adults were similar. Furthermore, three serotypes of hvKP (K5, K20, and K57) implicated in community-acquired human infections were also detected in urban rodents and shrews.

### Genotypes

Our samples were all ESBL-positive isolates, and PFGE profiles demonstrated high clonal dissemination of which in the humans and rodent animals. The higher discriminatory power of PFGE is consistent with the results of previous clinical isolates in the NICU or PICU and environmental samples [40, 41]. In our study, two isolates from shrews and healthy people, respectively, were clustered in a PFGE clustering tree. These two isolates were high-virulence of serotype K1 and ST23. The similarity of the two isolates was 88.4%. In the report by Lu, *et. al*, ST23 was a common type of detection, which 22 KP isolates were all from stool specimens of outpatients with diarrhea in Beijing [42]. ST23 and ST35 were detected from shrews and healthy adults in our study. In a previous study, ST37 type could be detected in the sputum and stool samples of diarrhea children [43]. ST37 genotype had the highest detection rate in rodent and human stool samples in our study. It might suggest that ST23, ST35 and ST37 KP isolates were on a wide range of hosts.

We observed that the resistance rate to cefotaxime was significantly higher than that to ceftazidime among ESBL-producing KP isolates both from small mammals and healthy adults. In analysis of ESBL genotypes, TEM, SHV, and CTX-M types were predominantly observed among KP isolates from HA- and CA-infections [25]. Researchers in China have previously identified that the predominant ESBL genotype in Beijing, Guangdong, and Hangzhou was the CTX-M type [44, 45], which preferentially exhibited powerful hydrolysis of cefotaxime compared to ceftazidime [46]. This may be a result of different hydrolysis effects with diverse ESBL genotypes.

## Conclusions

To the best of our knowledge, this is the first study investigating fecal carriage of KP in urban rodents and shrews worldwide. It is also the first report on the potential relationships among KP isolates from urban rodents, shrews, and healthy adults in the community. It is meaningful to pay attention to the high prevalence and antibiotic resistance of fecal KP from urban rodents and shrews and their impact on community-acquired infections. However, due to a small size of sample in our study, bigger sample size should be needed to provide more powerful evidence in the future study.

## Methods

### Ethics statement

The study protocol was approved by the Animal Ethics and Welfare Committee of the School of Public Health, Southern Medical University and adhered to the guidelines for the Rules for the Implementation of Laboratory Animal Medicine (1998) from the Ministry of Health, China. All surgical procedures were performed under anesthesia with diethyl ether in efforts to minimize suffering. Endangered or protected species were not involved in this study. Healthy adults participated after signing an informed consent form. After samples were collected, the animals were sent to the animal experimental center of southern medical university for conducting harmless treatment.

### Collection of samples, identification and antimicrobial susceptibility testing of *K. pneumoniae* clinical isolates

Small mammals were captured alive around houses and buildings monthly using rat-trap cages (Yue-zong Co Ltd.) in a community in Baiyun District of Guangzhou city in southern China, between May and September 2015. Some faecal samples were collected from the animals and others from healthy adults were consecutively collected in the Physical Examination Center of the third affiliated hospital of Southern Medical University in Guangzhou from May to September 2015. All stool samples were transported to the lab in transport medium at 4 °C. Subsequently, stool samples were soaked in 5 mL of the nutrient broth (Land Bridge, Beijing, China) and incubated overnight at 37 ± 1 °C for bacterial enrichment. The presumptive isolates were confirmed as KP using microbiological tests combined with amplification of the species-specific *khe* gene. Microbiological tests of the purified colonies included gram staining and biochemical testing. The *khe* gene was identified using PCR as described previously [47]. KP ATCC® 700603 and *E. coli* ATCC® 25922 were used as positive and negative controls, respectively, in each test protocol.

Antibiotic susceptibility testing of KP was conducted using the Kirby-Bauer disc diffusion method according to Clinical and Laboratory Standards Institute; 2018 guidelines (CLSI). A panel of 17 representative antimicrobial agents belonging to 11 different classes of antibiotics was used. Screening and phenotypic confirmatory tests for ESBL in KP were conducted according to CLSI guidelines, 2018. Results were interpreted according to CLSI. KP (ATCC® 700603) and *E. coli* (ATCC® 25922) were used as quality control strains.

### Capsular serotyping, detection of resistance genes and hypervirulent genes

PCR was used to detect the six common capsular serotype genes (including K1, K2, K5, K20, K54, and K57) [34, 48]. The reaction mixture was kept at 95 °C for 3 min, followed by 30 cycles of 94 °C for 40s, 58 °C for 40s, 72 °C for 1 min, and 72 °C for 7 min. The PCR products were visualized and analyzed by agarose gel electrophoresis and sequencing. All ESBL-producing KP isolates were screened for antimicrobial resistance genes (*bla*_TEM_, *bla*_SHV_, and *bla*_CTX-M_) by PCR as described previously [49–51]. Virulence genes, including *magA*, *rmpA*, *uge*, *kfu*, *wcaG*, and *aerobactin* among all hvKP isolates were screened by PCR using previously described methods [43, 52–56]. PCR primers and conditions have been described elsewhere (Additional file 2: Table S1, S2). All the positive products were sequenced and analyzed using the BLAST website (https://blast.ncbi.nlm.nih.gov/Blast.cgi) and the CARD website (https://card.mcmaster.ca/home).

### Molecular epidemiology

Genetic relatedness among the ESBL-producing KP isolates was determined by pulsed-field gel electrophoresis (PFGE). In brief, the cell suspension of *K. pneumoniae*, placed in plugs, which were made by adding an equal volume of molten 1.0% SeaKem Gold. Slices of *K. pneumoniae* plugs were digested with 45 U/slice XbaI (TaKaRa, Dalian, China) and incubated at 37 °C for 2 h. Electrophoresis was run on the CHEF-DRIII system (120° angle, 6 V/cm, switch times of 6 and 36 s). PFGE patterns were analyzed using the BioNumerics software package (version 5.10, Applied Maths, Inc., Austin, TX, USA). The similarity analysis was performed by Dice coefficient. A similarity of > 80% upon dendrogram analysis were considered to represent PFGE pattern subtypes [40]. A subset of isolates that represented the different PFGE clusters were further studied by multilocus sequence typing (MLST). Seven housekeeping genes (*rpoB, gapA, mdh, pgi, phoE, infB, tonB*) were detected according to the MLST official website (http://bigsdb.web.pasteur.fr/). Primer sequences, the annealing temperature, and amplified fragment sizes were shown in Additional file 2: Table S3. The reaction mixture was kept at 94 °C for 2 min, followed by 35 cycles of 94 °C for 20s, annealing for 30s, 72 °C for 30s, and 72 °C for 5 min.

### Statistical analysis

Statistical analysis was carried out by using SPSS 20.0 software. The chi-square test was used to evaluate if the prevalence of KP was significantly different among groups. A two-sided *p*-value of < 0.05 was considered to be statistically significant. EBURST v3.0 software was used to analyze our MLST results and database data.

## Supplementary information

**Additional file 1: Figure S1.** Tenmporal distribution of urban rodents and shrews between May and September, 2015.

**Additional file 2: Table S1.** Primer sequences of specific gene and resistant genes for *K. pneumoniae.* **Table S2.** Primer sequences of *K. pneumoniae* for hypervirulent serotypes and virulent genes. **Table S3.** Primer sequences of seven housekeeping genes of *K. peumoniae* for multilocus sequence typing. **Table S4.** Carriage rates of KP isolates from urban rodents, house shrews, and healthy people. **Table S5.** Prevalence of multidrug resistance and ESBL-production among KP isolates from rodents, shrews, and healthy people (%). **Table S6.** Detection of hypervirulent KP isolates and virulent genes from rodents, shrews, and healthy people.

## Data Availability

All data generated or analyzed during this study are included in this published article and its supplementary information files. Access to raw data can be acquired by connecting to the corresponding author via email.

## Abbreviations

CA: Community-acquired
CLSI: Clinical and Laboratory Standards Institute
ESBL: Extended-spectrum β-lactamases
I: Intermediate
KP: *K. pneumoniae*
KP: *Klebsiella pneumoniae*
MDR: Multidrug-resistant
MLST: Multilocus sequence type
PCR: Polymerase-chain reaction
PFGE: Pulsed-field gel electrophoresis
R: Resistant
S: Susceptible
