# Supplementary figures and images for: Comparisons of microbiological characteristics and antibiotic resistance of Klebsiella pneumoniae isolates from urban rodents, shrews, and healthy people
Source: BMC Microbiol. 2020 Jan 14;20:12. doi: 10.1186/s12866-020-1702-5 (PMC6961239; doi:10.1186/s12866-020-1702-5)

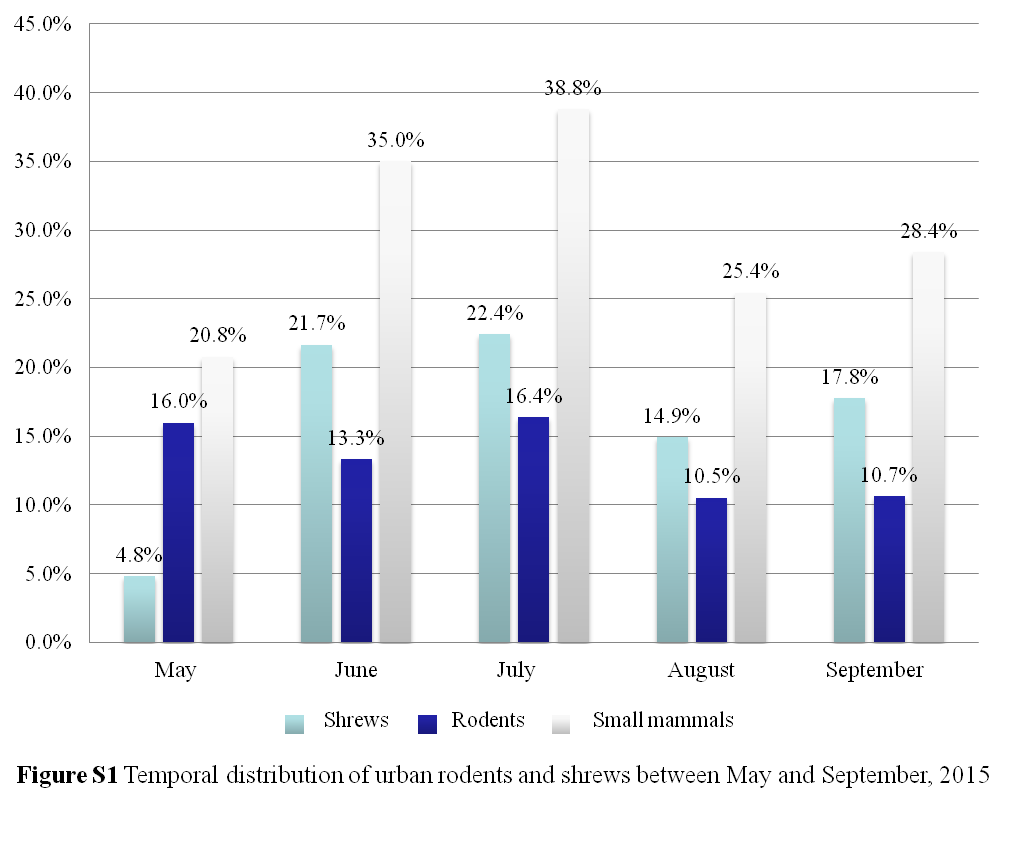

Supplement: Supplementary file 1 — Additional file 1: Figure S1. Tenmporal distribution of urban rodents and shrews between May and September, 2015. [file 12866_2020_1702_MOESM1_ESM.png]
