# Supplementary material for: Comparisons of microbiological characteristics and antibiotic resistance of Klebsiella pneumoniae isolates from urban rodents, shrews, and healthy people
Source: BMC Microbiol. 2020 Jan 14;20:12. doi: 10.1186/s12866-020-1702-5 (PMC6961239; doi:10.1186/s12866-020-1702-5)
Supplement: Supplementary file 2 — Additional file 2: Table S1. Primer sequences of specific gene and resistant genes for K. pneumoniae. Table S2. Primer sequences of K. pneumoniae for hypervirulent serotypes and virulent genes. Table S3. Primer sequences of seven housekeeping genes of K. peumoniae for multilocus sequence typing. Table S4. Carriage rates of KP isolates from urban rodents, house shrews, and healthy people. Table S5. Prevalence of multidrug resistance and ESBL-production among KP isolates from rodents, shrews, and healthy people (%). Table S6. Detection of hypervirulent KP isolates and virulent genes from rodents, shrews, and healthy people. [file 12866_2020_1702_MOESM2_ESM.docx]

**Supplemental Materials**

**
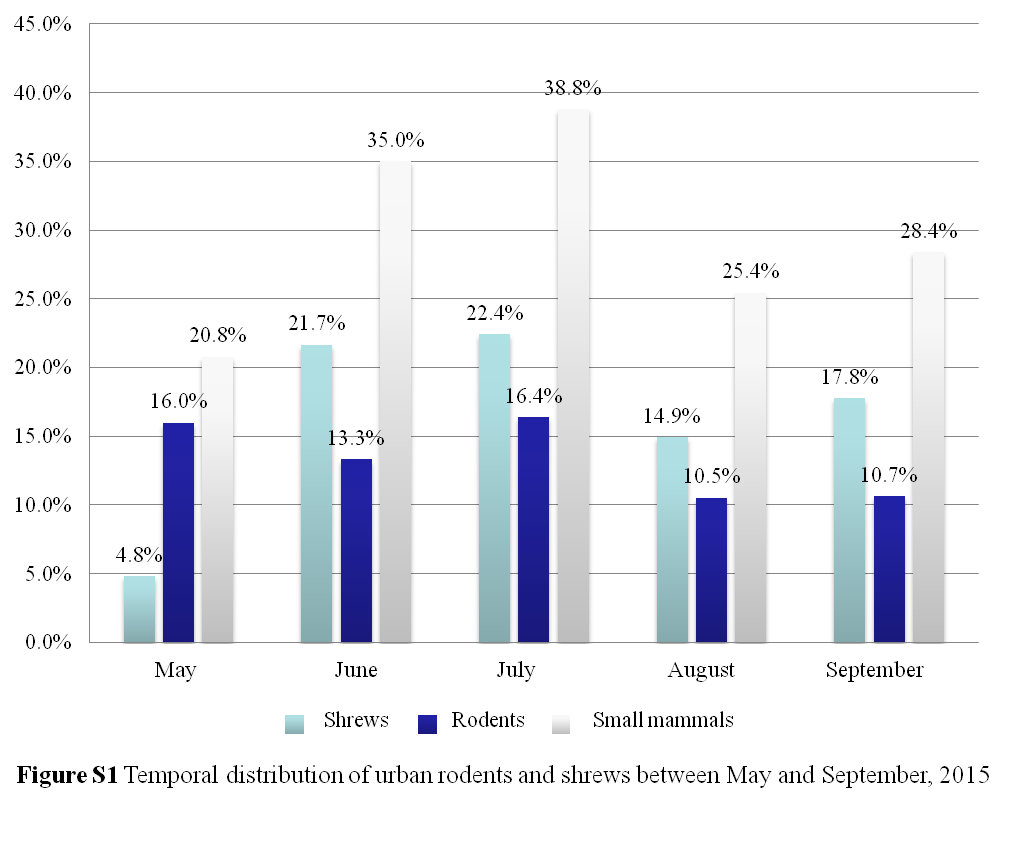
**

**Table S1 Primer sequences of specific gene and resistant genes for *K. pneumoniae***

| **Genes** | **Primers** | **Sequences (5’-3’)** | **Fragments (bp)** | **References** |
| --- | --- | --- | --- | --- |
| *khe* | khe-F | TGATTGCATTCGCCACTGG | 428 | ^[^[^1^](#_ENREF_1)^]^ |
|  | khe-R | GGTCAACCCAACGATCCTG |  |  |
| *bla*_TEM_ | TEM-F | ATGAGTATTCAACATTTCCG | 851 | ^[^[^2^](#_ENREF_2)^]^ |
|  | TEM-R | TTAATCAGTGAGGCACCTAT |  |  |
| *bla*_SHV_ | SHV-F | GCAAAACGCCGGGTTATTC | 940 | ^[^[^3^](#_ENREF_3)^]^ |
|  | SHV-R | GGTTAGCGTTGCCAGTGCT |  |  |
| *bla*_CTX-M_ | CTX-M-F | CGCTTTGCGATGTGCAG | 551 | ^[^[^4^](#_ENREF_4)^]^ |
|  | CTX-M-R | ACCGCGATATCGTTGGT |  |  |

**Table S2 Primer sequences of *K. pneumoniae* for hypervirulent serotypes and virulent genes**

| Genes | Primers | | Sequences (5ʹ-3ʹ) | | Fragments (bp) | | References |
| --- | --- | --- | --- | --- | --- | --- | --- |
| K1 | K1-F | | GTAGGTATTGCAAGCCATGC | | 1047 | ^[^[^5^](#_ENREF_5)^]^ | |
|  | K1-R | | GCCCAGGTTAATGAATCCGT | |  |  |  |
| K2 | *wzy*-K2-F | | GACCCGATATTCATACTTGACAGAG | | 641 | ^[^[^6^](#_ENREF_6)^]^ | |
|  | *wzy*-K2-R | | CCTGAAGTAAAATCGTAAATAGATGGC | |  |  |  |
| K5 | *wzx*-K5-F | | TGGTAGTGATGCTCGCGA | | 280 | ^[^[^6^](#_ENREF_6)^]^ | |
|  | *wzx*-K5-R | | CCTGAACCCACCCCAATC | |  |  |  |
| K20 | *wzy*-K20-F | | CGGTGCTACAGTGCATCATT | | 741 | ^[^[^5^](#_ENREF_5)^]^ | |
|  | *wzy*-K20-R | | GTTATACGATGCTCAGTCGC | |  |  |  |
| K54 | *wzx*-K54-F | | CATTAGCTCAGTGGTTGGCT | | 881 | ^[^[^5^](#_ENREF_5)^]^ | |
|  | *wzx*-K54-R | | GCTTGACAAACACCATAGCAG | |  |  |  |
| K57 | *wzy*-K57-F | | CTCAGGGCTAGAAGTGTCAT | | 1037 | ^[^[^5^](#_ENREF_5)^]^ | |
|  | *wzy*-K57-R | | CACTAACCCAGAAAGTCGAG | |  |  |  |
| *magA* | *magA*-F | | GGTGCTCTTTACATCATTGC | | 1280 | ^[^[^7^](#_ENREF_7)^]^ | |
|  | *magA*-R | | GCAATGGCCATTTGCGTTAG | |  |  |  |
| *rmpA* | *rmpA*-F | | ACTGGGCTACCTCTGCTTCA | | 535 | ^[^[^8^](#_ENREF_8)^]^ | |
|  | *rmpA*-R | | CTTGCATGAGCCATCTTTCA | |  |  |  |
| *uge* | *uge*-F | | TCTTCACGCCTTCCTTCACT | | 534 | ^[^[^9^](#_ENREF_9)^]^ | |
|  | *uge*-R | | GATCATCCGGTCTCCCTGTA | |  |  |  |
| *kfu* | *kfu*-F | | GAAGTGACGCTGTTTCTGGC | | 797 | ^[^[^10^](#_ENREF_10)^]^ | |
|  | *kfu*-R | | TTTCGTGTGGCCAGTGACTC | |  |  |  |
| *wcaG* | *wcaG*-F | | GGTTGGKTCAGCAATCGTA | | 169 | ^[^[^2^](#_ENREF_2)^]^ | |
|  | *wcaG*-R | | ACTATTCCGCCAACTTTTGC | |  |  |  |
| *aerobactin* | | *aero*-F | | GCATAGGCGGATACGAACAT | 556 | ^[^[^11^](#_ENREF_11)^]^ | |
|  |  | *aero*-R | | CACAGGGCAATTGCTTACCT |  |  |  |

**Table S3 Primer sequences of seven housekeeping genes of *K. peumoniae* for multilocus sequence typing**

| **Genes** | **Primers** | **Sequences (5ʹ-3ʹ)** | **Ta Opt** | **Fragments (bp)** |
| --- | --- | --- | --- | --- |
| *rpoB* | *rpoB*-F | GGCGAAATGGCWGAGAACCA | 50 | 501 |
|  | *rpoB*-R | GAGTCTTCGAAGTTGTAACC |  |  |
| *gapA* | *gapA-*F | TGAAATATGACTCCACTCACGG | 60 | 450 |
|  | *gapA*-R | CTTCAGAAGCGGCTTTGATGGCTT |  |  |
| *mdh* | *mdh*-F | CCCAACTCGCTTCAGGTTCAG | 50 | 477 |
|  | *mdh*-R | CCGTTTTTCCCCAGCAGCAG |  |  |
| *pgi* | *pgi*-F | GAGAAAAACCTGCCTGTACTGCTGGC | 50 | 432 |
|  | *pgi*-R | CGCGCCACGCTTTATAGCGGTTAAT |  |  |
| *phoE* | *phoE*-F | ACCTACCGCAACACCGACTTCTTCGG | 50 | 420 |
|  | *phoE*-R | TGATCAGAACTGGTAGGTGAT |  |  |
| *infB* | *infB*-F | CTCGCTGCTGGACTATATTCG | 50 | 318 |
|  | *infB*-R | CGCTTTCAGCTCAAGAACTTC |  |  |
| *tonB* | *tonB*-F | CTTTATACCTCGGTACATCAGGTT | 45 | 414 |
|  | *tonB-*R | ATTCGCCGGCTGRGCRGAGAG |  |  |

**Table S4. Carriage rates of KP isolates from urban rodents, house shrews, and healthy people**

| **Origin of sample** | **No. of sample** | **No. of KP** | **Carriage rate of KP (%)** |
| --- | --- | --- | --- |
| **Small mammals** | 190 | 149 | 78.42 |
| Rodents | 85 | 63 | 74.12 |
| *Rattus norvegicus* | 80 | 63 | 78.75 |
| *Mus musculus* | 3 | 0 | 0 |
| *Rattus flavipectus* | 2 | 0 | 0 |
| House shrews | 105 | 86 | 81.90 |
| *Suncus murinus* | 105 | 86 | 81.90 |
| **Healthy adults** | 275 | 182 | 66.18 |

**Table S5. Prevalence of multidrug resistance and ESBL-production among KP isolates from rodents, shrews, and healthy people (%)**

| **Prevalence of resistance** | **Rodents**  **(n=63)** | **Shrews**  **(n=86)** | **Healthy adults (n=182)** |
| --- | --- | --- | --- |
| Multidrug resistance | 49.21(31/63) | 36.04(31/86) | 47.80(87/182) |
| ESBLs-production | 7.94(5/63) | 12.79(11/86) | 17.03(31/182) |
| Resistance genes |  |  |  |
| *bla*_CTX-M_^a^ | 2/5 | 2/11 | 32.26(10/31) |
| *bla*_TEM_^c^ | 1/5 | 1/11 | 35.48(11/31) |
| *bla*_SHV_^c^ | 1/5 | 3/11 | 12.90(4/31) |

a: Two groups of gene types were detected: group 1 and group 9 of *bla*_CTX-M_.

b: Two gene types were detected: *bla*_TEM-1_.

c: Three gene types were detected: *bla*_SHV-27_, *bla*_SHV-40_ and *bla*_SHV-70_.

**Table S6. Detection of hypervirulent KP isolates and virulent genes from rodents, shrews, and healthy people**

| **Capsular serotype (No.)** | **Serotype (No.)** | | | **Virulence gene (No.)** | | | | | |
| --- | --- | --- | --- | --- | --- | --- | --- | --- | --- |
|  | **Rodents (n=63)** | **Shrews**  **(n=86)** | **Healthy adults (n=182)** | ***magA*** | ***rmpA*** | ***aerobactin*** | ***kfu*** | ***uge*** | ***wcaG*** |
| K1(3) | 0 | 1 | 2 | 3 | 2 | 2 | 2 | 3 | 3 |
| K2(3) | 0 | 0 | 3 | 0 | 3 | 3 | 0 | 3 | 0 |
| K5(6) | 1 | 4 | 1 | 0 | 0 | 0 | 3 | 6 | 0 |
| K20(2) | 1 | 0 | 1 | 0 | 1 | 1 | 1 | 2 | 1 |
| K54(1) | 0 | 0 | 1 | 0 | 0 | 0 | 0 | 1 | 1 |
| K57(5) | 1 | 1 | 3 | 0 | 1 | 1 | 2 | 3 | 1 |

**References:**

1. Yin-Ching C, Jer-Horng S, Ching-Nan L, Ming-Chung C: **Cloning of a gene encoding a unique haemolysin from *Klebsiella pneumoniae* and its potential use as a species-specific gene probe**. *Microbial pathogenesis* 2002, **33**(1):1-6.

2. Grimm V, Ezaki S, Susa M, Knabbe C, Schmid RD, Bachmann TT: **Use of DNA microarrays for rapid genotyping of TEM beta-lactamases that confer resistance**. *Journal of clinical microbiology* 2004, **42**(8):3766-3774.

3. Grobner S, Linke D, Schutz W, Fladerer C, Madlung J, Autenrieth IB, Witte W, Pfeifer Y: **Emergence of carbapenem-non-susceptible extended-spectrum beta-lactamase-producing *Klebsiella pneumoniae* isolates at the university hospital of Tubingen, Germany**. *Journal of medical microbiology* 2009, **58**(Pt 7):912-922.

4. Turton JF, Baklan H, Siu LK, Kaufmann ME, Pitt TL: **Evaluation of a multiplex PCR for detection of serotypes K1, K2 and K5 in *Klebsiella sp.* and comparison of isolates within these serotypes**. *FEMS microbiology letters* 2008, **284**(2):247-252.

5. Paterson DL, Hujer KM, Hujer AM, Yeiser B, Bonomo MD, Rice LB, Bonomo RA: **Extended-spectrum beta-lactamases in *Klebsiella pneumoniae* bloodstream isolates from seven countries: dominance and widespread prevalence of SHV- and CTX-M-type beta-lactamases**. *Antimicrobial agents and chemotherapy* 2003, **47**(11):3554-3560.

6. Nadasy KA, Domiati-Saad R, Tribble MA: **Invasive *Klebsiella pneumoniae* syndrome in North America**. *Clinical infectious diseases : an official publication of the Infectious Diseases Society of America* 2007, **45**(3):e25-28.

7. Fang CT, Lai SY, Yi WC, Hsueh PR, Liu KL, Chang SC: ***Klebsiella pneumoniae* genotype K1: an emerging pathogen that causes septic ocular or central nervous system complications from pyogenic liver abscess**. *Clinical infectious diseases : an official publication of the Infectious Diseases Society of America* 2007, **45**(3):284-293.

8. Kitchel B, Rasheed JK, Patel JB, Srinivasan A, Navon-Venezia S, Carmeli Y, Brolund A, Giske CG: **Molecular epidemiology of KPC-producing *Klebsiella pneumoniae* isolates in the United States: clonal expansion of multilocus sequence type 258**. *Antimicrobial agents and chemotherapy* 2009, **53**(8):3365-3370.

9. Fang CT, Chuang YP, Shun CT, Chang SC, Wang JT: **A novel virulence gene in *Klebsiella pneumoniae* strains causing primary liver abscess and septic metastatic complications**. *The Journal of experimental medicine* 2004, **199**(5):697-705.

10. Turton JF, Perry C, Elgohari S, Hampton CV: **PCR characterization and typing of *Klebsiella pneumoniae* using capsular type-specific, variable number tandem repeat and virulence gene targets**. *Journal of medical microbiology* 2010, **59**(Pt 5):541-547.

11. Yu VL, Hansen DS, Ko WC, Sagnimeni A, Klugman KP, von Gottberg A, Goossens H, Wagener MM, Benedi VJ: **Virulence characteristics of *Klebsiella* and clinical manifestations of *K. pneumoniae* bloodstream infections**. *Emerging infectious diseases* 2007, **13**(7):986-993.
